# S100P Expression in response to sex steroids during the implantation window in human endometrium

**DOI:** 10.1186/1477-7827-10-106

**Published:** 2012-12-07

**Authors:** Dan Zhang, Chengbin Ma, Xiaoli Sun, Hexia Xia, Wei Zhang

**Affiliations:** 1 Institute of Obstetrics and Gynecology Hospital, Fudan University, Shanghai, China; 2 Health Center for Women and Children, Chang ning district, Shanghai, China

**Keywords:** Calcium-binding protein S100P, Endometrial receptivity, Hormonal regulation

## Abstract

**Background:**

S100P, a protein originally detected in the human placenta, has been found to play an important role in the development and invasion of tumors. Interestingly, we have recently discovered using data mining that S100P was considerably up-regulated during the window of implantation in the human endometrium, but little further information has been available.

**Methods:**

Real-time PCR and immunofluorescence were performed to examine the expression and location of S100P in the human endometrium and endometrial cells. Estrogen and progesterone were added to the cultured cells to test the response of S100P to sex steroids.

**Results:**

A dramatic peak, approximately a 100-fold increase in comparison with the proliferative and early- and late-secretory phases, was observed in the endometrium during the mid-secretory phase, which corresponds to the time of embryo implantation. Progesterone regulated the expression of S100P in both primary endometrial epithelial and stromal cells, but estrogen had no significant effect.

**Conclusions:**

The results indicate that S100P participates in the periodic change of the endometrium under the regulation of progesterone, may be used as a unique biomarker of the receptive endometrium and play an important role in embryo implantation.

## Background

Embryo implantation is a bottleneck that limits successful pregnancy. A receptive endometrium and viable blastocyst are the two necessary conditions of successful implantation [1]. The endometrium is receptive only during the window of implantation, which lasts approximately four days (day 20–23), and occurs in humans during the mid-secretory phase in a normal 28-day menstrual cycle [2]. During the window of implantation, the endometrium undergoes extensive morphological and physiological changes to facilitate implantation of the embryo, including becoming more vascular and edematous with the glands displaying enhanced secretory activity [3]. This process is precisely regulated. Among all the regulating elements, reproductive hormones are the leading factors.

Recently, investigators have made great efforts to elucidate the molecular mechanisms of endometrial receptivity, and they have pursued biomarkers to identify the receptive endometrium. Previously, we employed bioinformatics to mine the existing microarrays and acquired a group of potential biomarkers [4], among them S100P attracted our attention.

S100P, originally isolated from the placenta, is a member of the family of S100 small molecular weight (9–14 kDa) calcium-binding proteins, which are mainly implicated in calcium sensing and signal transduction [5]. Recent studies have shown that S100P is highly expressed in many types of tumors [6-14] and influences proliferation, invasion, survival, metastasis, angiogenesis and resistance to chemotherapy drugs in a diverse group of tumors [14-19]. In our previous work using data mining, we found extremely high expression of S100P during the window of implantation, and a few other microarray studies have also shown the same up-regulation of S100P in LH + 7 or in the mid-secretory phase with a 6- to 20-fold change [20-22].

It has long been observed that there are strikingly similar biological processes between embryo implantation and tumor development and that the biological processes of proliferation, invasion, survival and angiogenesis are of crucial significance to a receptive endometrium and embryo implantation [23]. Thus, S100P might play an important role in the establishment of endometrial receptivity and embryo implantation.

The cyclical change of the endometrium is precisely controlled by reproductive hormones from the hypothalamus-pituitary-ovary axis. Studies of patients with premature ovarian failure (POF) have shown that E_2_ and P_4_ given sequentially can induce the establishment of endometrial receptivity, which indicates that E_2_ and P_4_ are the dominant factors as well as the only necessary factors that control the establishment of endometrial receptivity [24,25].

In a natural cycle, the granulosa cells of the developing follicle produce E_2_ in response to gonadotropin stimulation. Adequate E_2_ priming of the endometrium results in endometrial proliferation and the induction of sufficient P_4_ receptors to allow subsequent P_4_ stimulation for endometrial receptivity. In response to P_4_, the endometrium undergoes profound conformational and biochemical changes, from proliferative to secretory, with a concomitant induction of endometrial receptivity and the opening of the window of implantation. Steroid hormones act by regulating the expression of their downstream effectors [24].

Therefore, in the present study, we designed experiments to verify the expression and location of S100P in the human endometrium during the menstrual cycle, and we further investigated the regulation of S100P by E_2_ and P_4._

## Methods

### Sample collection

The endometrial samples (n = 24) were obtained via a pipelle catheter from fertile women, who provided informed consent under a protocol approved by the Committee of Fudan University Obstetrics and Gynecology Hospital on the Use of Human Subjects in Medical Research. Healthy volunteers or patients who were pathologically confirmed to have benign cervical lesions, aged 24 to 40, had regular cycles (27–33 d), and had not taken steroid hormone medications within 3 months were enrolled in the study.

Eighteen of the samples were kept on ice and were immediately transported to the laboratory. Some portions of the samples were routinely embedded in optimal cutting temperature (OCT) compound, and the frozen blocks were subjected to hematoxylin and eosin staining or immunofluorescence staining. The histological evaluation was blindly conducted by two independent pathologists. The specimens were classified according to the criteria of Noyes *et al*. [26] as proliferative phase (d8-14, n = 6), early-secretory phase (d15-18, n = 4), mid-secretory phase (d19-23, n = 5), and late-secretory phase (d24-28, n = 3). The other specimens were snap-frozen in liquid nitrogen for RNA and protein isolation.

Another six samples (mid- or late-proliferative phase) were transported to the laboratory for primary cell isolation.

### Culture of primary cells

The six samples were mixed together for cell isolation. Isolation of primary endometrial epithelial and stromal cells (EECs and ESCs) was performed as described previously [27] with some modifications. The endometrial tissues were minced (<0.1 mm^3^) before being digested via incubation in Dulbecco’s minimum essential medium (DMEM)/F-12 containing 0.25% type II collagenase (Sigma, St. Louis, MO) at 37°C for 1 h. The dispersed endometrial cells were separated into ESCs and EECs by filtration through a nylon cell strainer with 40-um pores. The filtered ESCs were centrifuged and plated in DMEM/F-12 containing 10% fetal bovine serum (Gibco, Australia) in 100-mm culture dishes. At the first passage, they were placed on 6-well dishes at a density of 5 × 10^5^ cells per well. Those that had reached confluence in 2 or 3 days were used for the experiments.

Unfiltered glands were rinsed with more than 30 ml of PBS before being back-flushed from the sieves. After centrifuging, the EECs were planted on 35-mm culture dishes at a density of 4 × 10^5^ cells per well using the same medium. Any ESCs remaining with EECs were further separated by selective adherence to culture dishes for 1 h. The cells that reached confluence in 2 or 3 days were used for the experiments. The purity of both stromal and epithelial cells was achieved by over 95%, as determined by cellular staining with vimentin and cytokeratin (See Additional file 1, Figure S1).

### Hormonal stimulation protocol

Cultured cells with a number of 1 × 10^5^ were placed in 1 ml of medium and seeded into a 12-well plate, and cultured cells with a number of 1 × 10^4^ were placed in 200 μl of medium and seeded into a 96-well plate. Both were subjected to 24-hour culture to achieve 50% confluence in normal medium containing 10% FBS. The half-confluent monolayers were maintained in serum-free media for 48 h prior to hormonal stimulation. We first treated the cells with 17beta-estradiol (Sigma, USA) at 10^-8^ mol/L, Progesterone (Sigma, USA) at 10^-7^ and E_2_ combined with P_4_ (10^-8^ mol/L E_2_ with 10^-7^ mol/L P_4_) for 48 h to test the response of S100P to reproductive hormones. And then we chose P_4_ and stromal cells to proceed the time and dose course experiments, treating stromal cells with different concentrations of P_4_ (10^-7^, 10^-8^ and 10^-9^ mol/L) for 48 h or P_4_ (10^-7^ mol/L) for different periods of time (24, 48 and 72 h). The hormonally treated cells that were seeded in 12-well plates were collected for real-time reverse transcriptase-polymerase chain reaction (real-time PCR) analysis, and those seeded in 96-well plates were fixed for in-cell Western analysis.

### RNA isolation and real-time PCR analysis

Total RNA was isolated using Trizol reagent (Invitrogen, USA) and the cDNA generated using the RevertAidTM First Strand cDNA Synthesis Kit (Fermentas, Canada) according to the manufacturer’s protocol.

Quantitative PCR reactions were carried out with SYBR Premix Ex Taq (Takara) and detected with an Applied Biosystem PRISM 7500HT system. The expression of S100P was normalized to that of the beta-actin in all samples. The primers were designed using the software Primer3 [28] as follows:

S100P, forward 5’-TACCAGGCTTCCTGCAGAGT-3’

reverse 5’-AGGGCATCATTTGAGTCCTG-3’;

beta-actin, forward 5’-CGGGACCTGACTGACTACTCA-3’

reverse 5’-TCAAGAAAGGGTGTAACGCAACTA -3’.

All experiments were performed in triplicate.

### Tissue immunofluorescence

Endometrial biopsies were embedded in optimum cutting temperature compound (Tissue-Tech, Sakura Finetechnical, Japan), snap-frozen, and cut into 5-μm thick sections. After fixation in cold acetone for 30 min, the sections were incubated with 5% bovine serum albumin (BSA) for 30 minutes at room temperature. Afterwards, they were incubated with mouse anti-human S100P (R&D, diluted 1:50 in phosphate-buffered saline PBS) for 2 h at 37°C. After three 10-minute washes in PBS, the sections were incubated with cy3-labeled goat anti-mouse IgG (Invitrogen) at 1:500 for 1 h at room temperature. The same washing procedures were performed, and the nuclei were counterstained with Hoechst33258 stain (Sigma) at 1 mg/ml. The cells were then observed under a fluorescence microscope (Axiovert 200, Zeiss, Germany).

### Cellular immunofluorescence staining

To further observe the intracellular location of S100P in the endometrial cells, immunofluorescence staining for S100P was performed on cell climbing slices. Cell climbing slices were placed on the bottom of 6-well dishes before seeding the first passage cells, which were maintained in DMEM/F-12 containing 10% fetal bovine serum. The slices were removed when the cells reached 50% confluence, and they were fixed in 3.7% formaldehyde for 20 min before being permeabilized and blocked using PBS containing 0.1% Triton X-100 (Sigma) and 5% BSA at 37°C for 45 min. The slices were incubated overnight at 4°C with 1:50 mouse anti-human S100P monoclonal antibody (R&D Systems, Minneapolis, MN) diluted in blocking buffer. They were washed three times in PBS for 5 min and incubated with 1:500 cy3-labeled goat anti-mouse IgG (Invitrogen) for 1 h at room temperature. The same washing procedures were performed again, and the nuclei were counterstained with Hoechst33258 stain (Sigma) at 1 mg/ml. The cells were observed under the same fluorescence microscope (Axiovert 200, Zeiss, Germany).

### In-cell western

In-cell Western, an immunocytochemical assay to quantify proteins in fixed cells [29], was performed to evaluate the protein level changes of S100P after hormonal treatment. The cells were seeded in 96-well plates, and treatments were performed as described above. The medium was removed, the cells were fixed in 200 μl of 3.7% formaldehyde in PBS for 20 min, and they were permeabilized and blocked using 200 μl PBS containing 0.1% Triton X-100 and 5% BSA at 37°C for 45 min. Afterwards, they were incubated overnight at 4°C with 1:50 mouse anti-human S100P monoclonal antibody (MAB2957, R&D Systems) and 1:200 rabbit anti-human beta-actin antibody (1854–1, Epitomics, USA) diluted in blocking buffer. They were washed three times with PBS for 5 minutes, and the dishes were incubated with 1:1000 fluorescently labeled IRDye 800 anti-mouse and IRDye 680 anti-rabbit secondary antibodies (KPL, USA) for 1 h at room temperature, followed by the same washing procedures. The negative controls were obtained by omitting the primary antibodies. The intensity of cellular staining was quantified by densitometry using an Odyssey infrared imaging system (Li-Cor Biosciences). The specificity of the primary antibodies has been validated by Western blot prior to the experiments (See Additional file 2, Figure S2).

### Statistical analysis

The results are presented as the means ± SD of three to six independent experiments. Statistical analyses were performed using one-way ANOVA, followed by LSD analysis using SPSS 15.0 software, and their values were considered statistically significant at *P* < 0.05. All experiments were repeated at least three times.

## Results

### The mRNA expression of S100P in human endometrium

**Figure 1:**
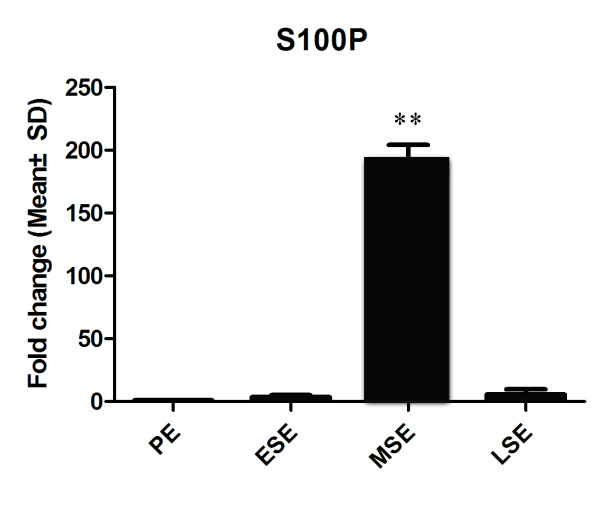
**Expression level of S100P mRNA.** The mean fold changes of S100P mRNA expression in human endometrium are shown; the four groups (PE, ESE, MSE, and LSE) contained 6, 4, 5, and 3 samples, respectively; the data was normalized to that of PE; and beta-actin was used as the housekeeping gene. PE: proliferative endometrium, ESE: early-secretory endometrium, MSE: mid-secretory endometrium, LSE: late-secretory endometrium (** *P* < 0.01).

The results of real-time PCR analysis showed that the mRNA expression of S100P was significantly up-regulated, with a peak in the mid-secretory endometium (MSE). The mRNA level in the MSE was 180 times as high as that in the proliferative endometrium (PE), 90 times as high as that in the early-secretory endometium (ESE) and over 70 times as high as that in the late-secretory endometium (LSE) (*P* < 0.01) (Figure 1).

### The protein expression and tissue location of S100P in human endometrium

**Figure 2:**
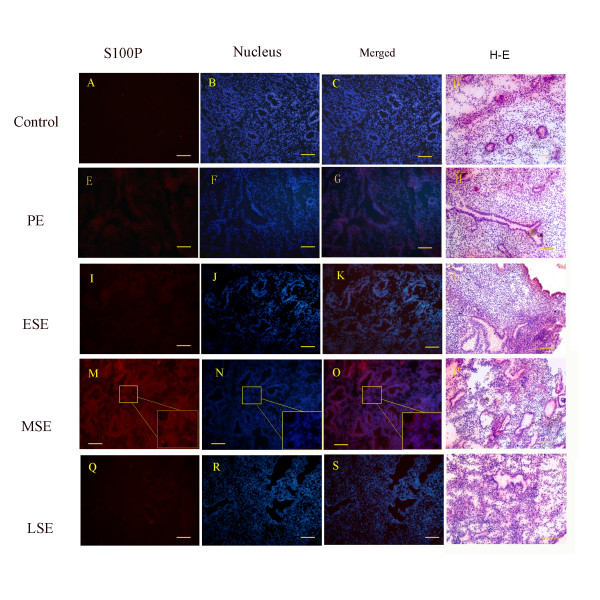
**Expression level and tissue distribution of S100P protein.** Immunofluorescence staining of S100P in human endometrium at PE (**E**, **F** and **G**), ESE (**I**, **J** and **K**), MSE (**M**, **N** and **O**), and LSE (**Q**, **R** and **S**) are shown; PBS substituted for the primary antibody was used as the negative control (**A**, **B** and **C**); Hoechst was used for nuclei staining (blue) (**B**, **F**, **J**, **N** and **R**); S100P fluorescent images (red) (**A**, **E**, **I**, **M** and **O**), merged images (**C**, **G**, **K**, **O** and **S**), and hematoxylin and eosin (HE) staining (**D**, **H**, **L**, **P** and **T**) are shown; higher magnification view of the selected areas in MSE staining (**M**, **N** and **O**) are shown in the lower right corner; scale bars represent 100um.

Immunofluorescence was performed to detect the protein expression and location of S100P in the endometrium during the menstrual cycle. In comparison with the results of real-time PCR, the S100P protein expression presented the same moving pattern, where S100P signals were nearly undetected in the PE, slightly elevated in the ESE, dramatically enhanced in the MSE and sharply weakened in the LSE. In the MSE, the enhanced S100P signals were distributed in the glands and the stroma, but the highest immunoreactivity was located in the glands (Figure 2).

### The cellular location of S100P in endometrial cells

**Figure 3:**
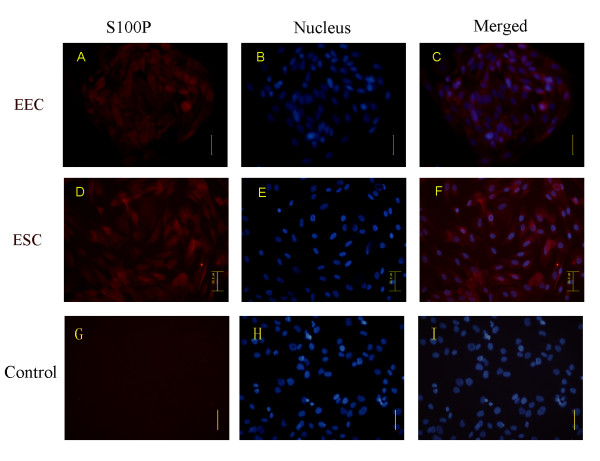
**Cellular location of S100P protein.** Immunofluorescence staining of S100P in EEC (primary endometrial epithelial cells) (**A**, **B** and **C**) and ESC (primary endometrial stromal cells) (**D**, **E** and **F**); PBS substituted for the primary antibody was used as the negative control (**G**, **H** and **I**); Hoechst was used for nuclei staining (blue) (**B**, **E** and **H**), S100P fluorescent images (red) (**A**, **D** and **G**), and merged images (**C**, **F** and **I**); the scale bars represent 50 μm.

The cellular immunofluorescence staining was conducted to observe the intracellular distribution of S100P. In the cultured primary endometrial epithelial and stromal cells, S100P was expressed in both the cytoplasm and the nucleus, although the signal intensity showed no significant difference between the cytoplasm and the nucleus (Figure 3).

### Regulation of S100P by reproductive hormones in human endometrium

**Figure 4:**
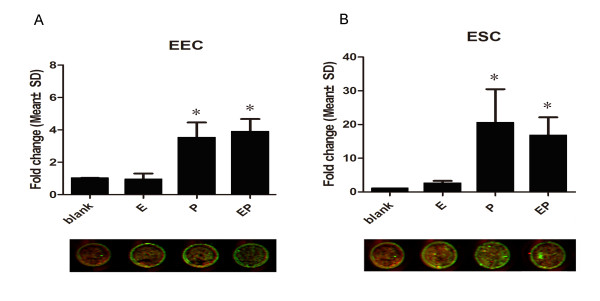
**Hormonal regulation of S100P.** The variations of S100P under hormonal stimulations in primary endometrial cells (A: EEC, B: ESC). The upper column graph shows the mRNA changes of S100P detected by real-time PCR; the lower panel indicats the protein level variations of S100P detected by in-cell Western. The data were normalized to that of PE and used beta-actin as the internal reference. E: estrogen, P: progesterone, EP: estrogen combined with progesterone, blank: unstimulated.

**Figure 5:**
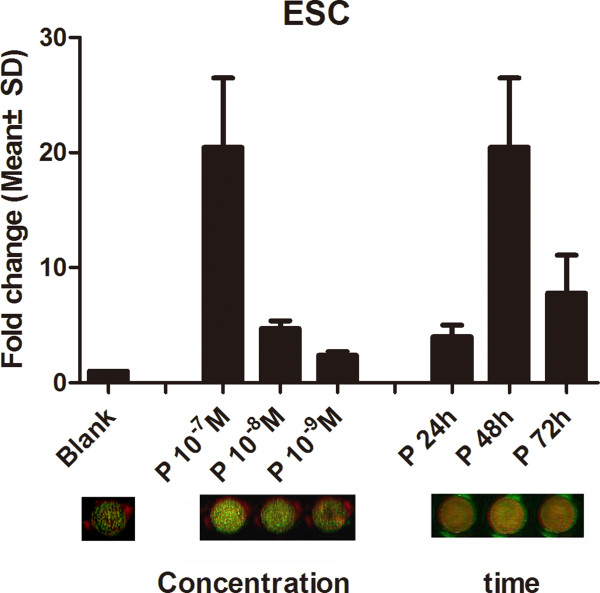
**Time course and dose-dependent reactions of S100P to hormonal stimulations in ESC.** The time course and dose-dependent reaction of S100P under the P_4_ stimulations tested in ESC are shown: the upper column graph shows the mRNA changes of S100P detected by real-time PCR; the lower panel indicates the protein level variations of S100P detected by in-cell Western. The data was normalized to that of unstimulated and used beta-actin as the internal reference. P: progesterone, blank: unstimulated.

We first observed the hormonal effects on S100P after 48 h of stimulation with the highest physiological dose (E_2_, 10^-8^ mol/L and P_4_, 10^-7^ mol/L). In the primary epithelial and stromal cells, the expression of S100P was found to be strongly stimulated by P_4_, and its expression rose 4-fold in EEC and 20-fold in ESC at both the mRNA and protein level (*P* < 0.001). E_2_ alone showed no obvious effects, and E_2_ combined with P_4_ did not display marked synergistic effects (Figure 4). Then, we further studied the time and dose effect of P_4_ on S100P in primary stromal cells. The expression of S100P peaked after stimulation for 48 h, and was maximized at 10^-7^ mol/L. (Figure 5).

## Discussion

In the present study, we determined the peak expression of S100P during the implantation window in terms of both the mRNA and protein levels, which was approximately 100 times high as those in the other phases of endometrium during the menstrual cycle. On the one hand, it suggests that S100P could be chosen as a unique biomarker of receptive endometrium. Moreover, the most prominent S100P signal in the MSE were located in the glands, which was not in accordance with the expression pattern found by Tong *et al*. [30], who showed that S100P is distributed in the nuclei and observed more intense S100P immune staining in the stroma compared to that in the glands. However, our results were similar to those for the gastric mucosa reported by Parkkila *et al*., who reported that the highest immunoreactivity was located in the surface epithelial cells and glands [31], suggesting that S100P has the potential to be secreted into the uterine cavity and detected in the uterine fluid as a non-invasive biomarker of endometrial receptivity. On the other hand, it suggests that S100P could be functionally related to the establishment and withdrawal of endometrial receptivity, the secretory activity of the endometrium and the maintenance of the inter-uterine environment. It has been reported that the expression of S100P increased 37-fold in stromal cells after their co-culture with trophoblast cells [32], which proved that S100P might be involved in interactions at the maternal-fetal interface.

Previous studies have shown that S100P can be localized in the cytoplasm and/or the nucleus of a wide range of cells, and as a secretory protein, it can also be secreted to extracellular regions [13,31,33]. In the present study, we observed that S100P is expressed in the nucleus as well as in the cytoplasm of endometrial cells and that it largely accumulated in the cytoplasm of EECs and ESCs. The cellular location of S100P has been reported to correspond to its molecular functions as follows: the intracellular component of S100P could interact with Ezrin, a multi-domain link protein of the membrane-cytoskeleton, thus playing a role in cell differentiation, adhesion, and migration [34]; S100P in the cytoplasm could bind to CacyBP/SIP, a component of the ubiquitin pathway, which is involved in the degradation of the cell signaling molecule ß-catenin [35]; the extracellular-soluble form of S100P could function as a ligand of the RAGE receptor to modulate cell proliferation and survival via activation of MAP kinase and NF-kappa B pathway [10]; and the component of S100P in the nucleus might bind to S100PBP (S100P binding protein), the exact role of which remains unclear.

It has been shown that cell proliferation, adhesion and motility are all vital to endometrial receptivity and embryo implantation, in which ß-catenin [36], MAP kinase [37] and the NF-kappa B pathway [38] play an important role. Ezrin, which regulates the reorganization of cytoskeleton-membrane connections [39], has also been shown to be involved. These findings suggest that S100P might act as a key linker participating in the formation of endometrial receptivity and embryo implantation.

Elucidating the regulation of S100P is an important step towards understanding the biological significance of and proposing strategies for targeted S100P modulation [14]. Recent studies have shown that DNA methylation [40], IL-6 [41], bone morphogenic protein [42], prostaglandin E (PGE)/EP4 [43], glucocorticoid [44], and non-steroidal anti-inflammatory drugs [45] could regulate S100P expression during tumor progression. Steroid hormones were also reported to play a role in the expression of S100P, including the synthetic androgen R1881, which regulates the expression of S100P in prostate cancer [46], as well as P_4_ and six other types of clinically relevant synthetic progestins, which up-regulate S100P in progesterone receptor (PR)-positive cell lines of breast cancer [47].

In the present study, we verified that P_4_ can up-regulate the expression of S100P in primary endometrial cells. The results of the time course experiments showed a parabolic shape, with a peak at 48 h, after which a sharp drop was observed. The dose-dependent experiments showed that S100P expression increased with increasing P_4_ concentrations within a certain range. This response explained the special temporal expression of S100P during the window of implantation resulting from the influence of P_4_. The level of progesterone secretion may have peaked at the mid-secretory phase in concert with the peak value of S100P expression, and the expression of S100P may have declined significantly with the time extension of progesterone at the late-secretory phase, although the concentration of progesterone decreased little.

## Conclusions

For the first time, we systematically clarified the expression and hormonal regulation of S100P in endometrial cells, and we found that its expression peaked during the window of implantation and that progesterone could function as effective regulators of S100P expression. The results indicate that S100P participates in periodic changes of the endometrium under the regulation of progesterone, that it might perform an important function in regulating endometrial development during the menstrual cycle and in developing conditions necessary for embryo implantation, and that it might be used as a unique biomarker of a receptive endometrium.

## Competing interests

The authors declare that they have no competing interests with respect to the authorship and/or publication of this article.

## Authors’ contributions

DZ participated in the design of the study, collected the materials, carried out all experiments, and drafted the manuscript. DZ, HX, and XS collected the materials and helped maintain the cell culture. CM and WZ conceived of the study. WZ participated in its design and coordination and helped draft the manuscript. All authors read and approved the final manuscript.

## Supplementary Material

Additional file 1 **Figure S1.** The purity staining of EECs and ESCs. Cell immunocytochemical characterization of EECs and ESCs are shown. Immunocytochemistry shows that EECs express cytokeratin 7 (C) but do not express vimentin (E), and ESCs express vimentin (F) but do not express cytokeratin 7 (D). PBS substituted for the primary antibody was used as the negative control (A and B). Scale bar = 50 um. (TIFF 1409 kb) Click here for file

Additional file 2 **Figure S2.** The specificity validation of the primary antibodies. The mouse anti-human S100P monoclonal antibody (1:1000) and rabbit anti-human beta-actin antibody (1:3000) were used as the primary antibody in Western blot analysis. Total 40 μg protein of each column was used. The upper panel shows the S100P signal detected from negative (A and B) and positive (C and D) controls, and the lower panel shows the equal of loading samples stained by beta-actin antibody. (TIFF 45 kb) Click here for file
