# Supplementary figures and images for: S100P Expression in response to sex steroids during the implantation window in human endometrium
Source: Reprod Biol Endocrinol. 2012 Dec 7;10:106. doi: 10.1186/1477-7827-10-106 (PMC3551790; doi:10.1186/1477-7827-10-106)

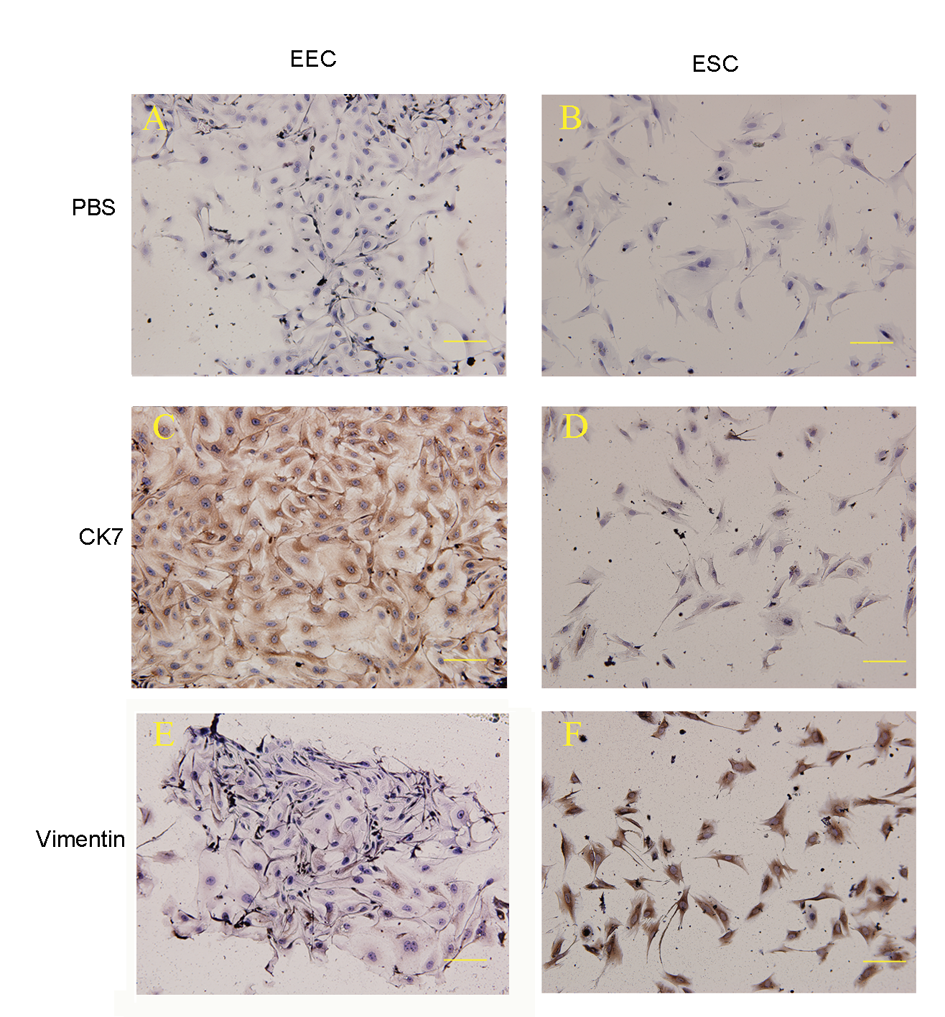

Supplement: Additional file 1 — Figure S1. The purity staining of EECs and ESCs. Cell immunocytochemical characterization of EECs and ESCs are shown. Immunocytochemistry shows that EECs express cytokeratin 7 (C) but do not express vimentin (E), and ESCs express vimentin (F) but do not express cytokeratin 7 (D). PBS substituted for the primary antibody was used as the negative control (A and B). Scale bar = 50 um. (TIFF 1409 kb) [file 1477-7827-10-106-S1.tiff]

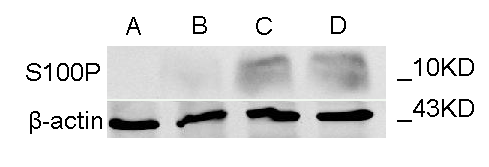

Supplement: Additional file 2 — Figure S2. The specificity validation of the primary antibodies. The mouse anti-human S100P monoclonal antibody (1:1000) and rabbit anti-human beta-actin antibody (1:3000) were used as the primary antibody in Western blot analysis. Total 40 μg protein of each column was used. The upper panel shows the S100P signal detected from negative (A and B) and positive (C and D) controls, and the lower panel shows the equal of loading samples stained by beta-actin antibody. (TIFF 45 kb) [file 1477-7827-10-106-S2.tiff]
